# Engineering a 3D Biomimetic Peptides Functionalized-Polyethylene Glycol Hydrogel Model Cocultured with Endothelial Cells and Astrocytes: Enhancing In Vitro Blood–Brain Barrier Biomimicry

**DOI:** 10.1021/acs.molpharmaceut.4c00599

**Published:** 2024-08-12

**Authors:** Nesrine Ahmad, Georges Kiriako, John Saliba, Kawthar Abla, Marwan El-Sabban, Rami Mhanna

**Affiliations:** † Biomedical Engineering Program, Maroun Semaan Faculty of Engineering and Architecture, American University of Beirut, Beirut 1107−2020, Lebanon; ‡ Department of Anatomy, Cell Biology and Physiological Sciences, Faculty of Medicine, American University of Beirut, Beirut 1107−2020, Lebanon

**Keywords:** 3D in vitro models, blood−brain barrier, fibronectin-mimetic RGD, laminin-mimetic IKVAV, polyethylene glycol, hydrogels

## Abstract

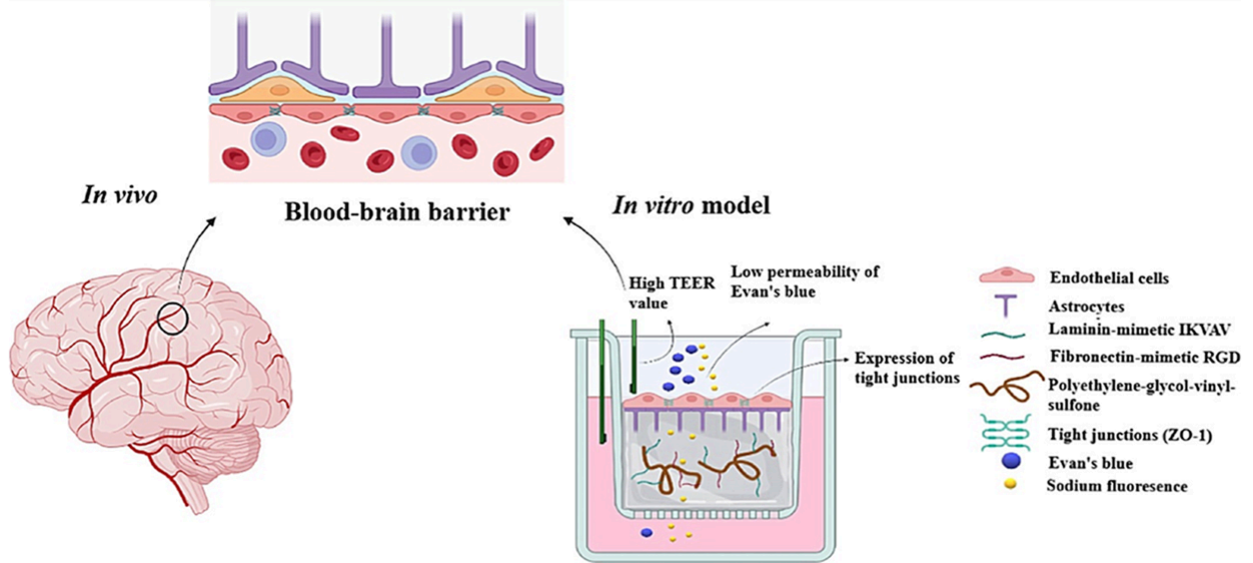

The blood–brain barrier (BBB) poses a significant challenge for drug delivery and is linked to various neurovascular disorders. In vitro BBB models provide a tool to investigate drug permeation across the BBB and the barrier’s response to external injury events. Yet, existing models lack fidelity in replicating the BBB’s complexity, hindering a comprehensive understanding of its functions. This study introduces a three-dimensional (3D) model using polyethylene glycol (PEG) hydrogels modified with biomimetic peptides that represent recognition sequences of key proteins in the brain. Hydrogels were functionalized with recognition sequences for laminin (IKVAV) and fibronectin peptides (RGD) and chemically cross-linked with matrix metalloprotease-sensitive peptides (MMPs) to mimic the extracellular matrix of the BBB. Astrocytes and endothelial cells were seeded within and on the surface of the hydrogels, respectively. The barrier integrity was assessed through different tests including transendothelial electrical resistance (TEER), the permeability of sodium fluorescence (Na–F), the permeability of Evan’s blue bound to albumin (EBA), and the expression of zonula occluden-1 (ZO-1) in seeded endothelial cells. Hydrogels with a combination of RGD and IKVAV peptides displayed superior performance, exhibiting significantly higher TEER values (55.33 ± 1.47 Ω·cm^2^) at day 5 compared to other 2D controls including HAECs-monoculture and HAECs-cocultured with NHAs seeded on well inserts and 3D controls including RGD hydrogel and RGD-IKVAV monoculture with HAECs and RGD hydrogel cocultured with HAECs and NHAs. The designed 3D system resulted in the lowest Evan’s blue permeability at 120 min (0.215 ± 0.055 μg/mL) compared to controls. ZO-1 expression was significantly higher and formed a relatively larger network in the functionalized hydrogel cocultured with astrocytes and endothelial cells compared to the controls. Thus, the designed 3D model effectively recapitulates the main BBB structure and function in vitro and is expected to contribute to a deeper understanding of pathological CNS angiogenesis and the development of effective CNS medications.

## 1 Introduction

Neurodegenerative diseases encompass a wide range of clinical and pathological conditions, such as Parkinson’s disease, Alzheimer’s disease, and Huntington’s disease. Advancing age is a significant risk factor for these diseases, and with the aging population, the World Health Organization projects a threefold increase in affected individuals over the next three decades.^1^ Yet, effective clinical interventions remain limited. One of the primary challenges in treating these diseases is the presence of the blood–brain barrier (BBB), a complex and dynamic interface consisting of endothelial cells, tight junctions (TJs), pericytes, and astrocytic endfeet.^2^ The physical and chemical composition of the BBB maintains brain homeostasis under physiological conditions, by preventing foreign and harmful molecules from entering the brain, posing a significant barrier to drug delivery for brain disorders.^3^

Given the persistence of brain diseases and the dire need for therapy improvements, developing physiologically relevant models that accurately mimic the BBB is crucial for understanding its selectivity and complexity in drug passage. While animal models closely recreate the physiological aspects of the BBB, such techniques are time-consuming and costly and present ethical concerns for effective drug development only based on in vivo models. Moreover, new regulations signed in December 2022 allow the FDA to rely on animal-free alternatives before human trials for drug development, creating an urgent need for advanced in vitro models that can recapitulate BBB key elements.^4^ Earlier studies relied on two-dimensional (2D) models, where endothelial cells are typically seeded on permeable transwell inserts rather than directly on solid plastic. In this setup, the interior of the inset simulates the luminal (blood) side, while the surrounding well, into which the inset is placed, represents the abluminal (brain) side. While the barrier integrity of such models can be enhanced through coculture with pericytes, astrocytes, and other neurovascular unit (NVU) cells, 2D models fail to fully reproduce the human BBB’s complexity due to the linear structure that limits cell–cell interactions, leading to inaccurate in vivo permeability estimates.^5^

Robust, and physiologically relevant microvascular models that mimic physiological and pathological features of the BBB are needed to boost the bench-to-bedside clinical translation of potential drugs. Consequently, it is crucial to develop in vitro three-dimensional (3D) platforms that better replicate the BBB’s structure and functions. 3D models, such as hydrogels, spheroids, organoids, and microfluidic-based BBB-on-a-chip models, have been engineered and offer promising alternatives. In general, 3D models are composed of either endothelial cell monocultures or cocultures with pericytes and/or astrocytes, embedded within a supporting matrix that mimics the brain’s extracellular matrix (ECM). These models self-assemble in suspension within a culture medium or within specific 3D structures that contain hollow channels that simulate cerebral blood capillaries. This allows them to effectively mimic the native tissue architecture and functionality.^4^ Recent studies in modeling BBB have focused on 3D systems. One study developed a 3D Matrigel coculture model with an immortalized human brain endothelial cell line and human astrocytes, showing high barrier integrity.^6^ However, the complexity and animal origin of Matrigel limits its clinical applications.^7^ Another study used a similar approach but employed a 3D collagen hydrogel matrix instead.^8^ This model demonstrated promising results for the small molecules’ movement across brain endothelium, yet had poor barrier properties and deficient protein expression compared to physiological conditions.^9^

In this study, immortalized human aortic endothelial cells (HAECs), isolated from the human ascending and descending aorta, and Normal Human Astrocytes (NHAs), isolated from the cerebral cortex of the human brain, were selected for their ability to mimic BBB characteristics. HAECs have been previously used to study endothelium dysfunction and develop effective treatments due to their ability to modulate cellular adhesion and interact with astrocytes to form TJ-like structures and filamentous actin, mimicking in vivo physiological conditions.^10^ Astrocytes are also important components of the human central nervous system. Their diverse and heterogeneous nature enables them to perform a variety of crucial functions during brain development and subsequently in maintaining neuronal homeostasis and facilitating synaptic transmission. NHAs play a crucial role in BBB maintenance by secreting promotive factors, such as transforming growth factor-β (TGFβ) and glial cell line-derived neurotrophic factor (GDNF), which lead to the adequate association between the BBB cells and the formation of strong TJs.^10^ Therefore, selecting appropriate cells and biomaterials that accurately mimic the ECM can be integrated into various models of the NVU to investigate the BBB function.

Polyethylene glycol (PEG) hydrogels, functionalized with laminin and fibronectin-mimetic peptides, IKVAV and RGD, respectively, were chosen as scaffolds for coculturing endothelial cells with astrocytes. PEG is an inert, biocompatible, biodegradable, flexible, tunable, hydrophilic, porous, and inexpensive material that has demonstrated potential in the field of tissue engineering and drug delivery.^11^ The 4-arm PEG-vinyl sulfone (PEG-VS) polymer is particularly advantageous due to its hydrolytic stability, rapid gelation, successful cell encapsulation, and modifiability with biological motifs.^12^ In addition, laminin and fibronectin are crucial for the development and regulation of the BBB by promoting TJ protein synthesis as well as cell growth and adhesion.^13^

This study aims to engineer a 3D in vitro BBB model that closely resembles the structure and functions of the human BBB by coculturing HAECs and NHAs in functionalized PEG hydrogels with IKVAV and RGD. To our knowledge, this combination has not been previously attempted. The 3D model’s biocompatibility was assessed using live/dead assays, its integrity was evaluated by measuring transendothelial electrical resistance (TEER), and its permeability through immunofluorescence assays of TJ proteins.

## 2 Materials and Methods

### 2.1 Materials

The 4-arm PEG-VS (MW 20,000) was obtained from JenKem Technologies, China. MMP-sensitive peptides (MW 2699.16), IKVAV peptides (MW 1007.22), and RGD Peptides (MW 346.34) were obtained from GL Biochem Shanghai, China. Phosphate buffered saline (PBS) solution 10× was purchased from Lonza, Switzerland. Fetal bovine serum (FBS), trypsin solution 10×, Tween-20, penicillin-streptomycin, Roswell Park Memorial Institute Medium (RPMI-1640 Medium), Dulbecco’s Modified Eagle’s Medium-High Glucose (DMEM-High Glucose), bovine serum albumin (BSA A9418–50G), Ethidium Homodimer (EthD), Paraformaldehyde (PFA), Fluorescein Sodium Salt, and Evan’s Blue were obtained from Sigma-Aldrich, USA. Astrocyte Basal Medium (ABM) was obtained from iXCells Biotechnologies, USA. Calcein-AM was received from Molecular Probes, USA. Mouse Anti-GFAP antibody was obtained from Abcam, UK. Rabbit Anti-ZO-1 antibody, Alexa Fluor 488 goat antirabbit IgG, Texas Red goat anti-mouse IgG, and DAPI were obtained from Thermofisher Scientific, USA. Triethanolamine (99%+; MW 149.19) was obtained from Arcos Organics, USA. Normal goat serum (NGS,S26–100 mL) was purchased from Chemicon International, USA. All the other solvents and chemicals used in the current investigation were of analytical grades.

### 2.2 Fabrication of PEG Hydrogel

PEG hydrogels were synthesized by a Michael-type addition reaction of thiol-containing peptides and 4-armed PEG-VS. In brief, different concentrations of PEG (3, 4, 5, and 6% w/v) in triethanolamine (TEOA) in which adhesion peptides (RGD/IKVAV), at a certain concentration, were mixed with 4% PEG-VS and then vortexed for 30 s. After incubating this mixture for 15 min at 37 °C and 5% CO_2_ at 37 °C, MMP-sensitive peptides (10%), acting as cross-linkers, were added to the functionalized PEG to bring the final molar ratio of VS:SH to 1:1. The cross-linked and functionalized PEG was vortexed for 5–10 s and pipetted into the corresponding membrane. The hydrogels were then incubated at 37 °C for 10 min to polymerize and become viscous. To retain moisture, a sufficient amount of complete medium was added to the hydrogels and then stored in the incubator at 37 °C and 5% CO_2_.^14^

The Young’s moduli of PEG hydrogels at different concentrations were characterized. Compressive moduli of the swelled hydrogels were measured using a universal mechanical testing device (Instron 5900 series, US). Samples were compressed at a speed of 0.01 mm/s, and Young’s moduli were calculated according to Hooke's Law:
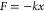
where *F* is the force, *x* is the length of extension/compression, and *k* is a constant of proportionality known as the spring constant, which is usually given in N/m.

### 2.3 Seeding of Astrocytes and Endothelial Cells

The study utilized the NHAs cell line and immortalized HAECs for all in vitro experiments. NHAs were cultured in ABM complete medium supplemented with 10% FBS and 1% antibiotics on PLL-coated T-25 flasks. HAECs were cultured in RPMI complete medium supplemented with 10% FBS and 1% penicillin-streptomycin on T-25 flasks. Both cell types were incubated at 37 °C/5% CO_2_ until the desired confluency was reached, then subcultured and collected at the desired cell count.

Before cross-linking the functionalized PEG with 10% MMP, NHAs were encapsulated within the PEG-peptide mixture in which the PEG was of 4% concentration at 10,000 cells/10 μL hydrogel cell density. The resulting hydrogel scaffolds were pipetted onto PLL-coated 12-well inserts and incubated for 10 min to promote gelation. ABM complete medium was added to the insets and wells. On day 4, HAECs were seeded on the hydrogel scaffolds at 500,000 cells/insert density and incubated at 37 °C/5% CO_2_ to form a 2D monolayer. RPMI complete medium was added and changed every 24 h for the insets and every 48 h for the wells.

### 2.4 Viability and Live/Dead Assay

To identify the optimal PEG and peptide concentration for the growth and spreading of astrocytes, a live/dead assay was conducted on days 1, 4, and 7 postfabrication. NHAs encapsulated within PEG hydrogels were functionalized with varying concentrations of IKVAV (75, 100, 300, or 600 μM) and/or RGD (200, 400, 600, or 800 μM) and stained with Calcein-AM and EthD-1 dyes. DMEM incomplete medium containing Calcein-AM and EthD-1 was added to the fabricated PEG hydrogel and incubated for 30 min at 37 °C and 5% CO_2_. After 30 min, the dye was removed, and samples were washed twice with PBS to remove any traces of the dye. Samples were then fixed with 4% PFA and stored in the dark at room temperature. After 25 min, the 4% PFA was removed, and samples were washed with PBS. Acellular PEG hydrogels were used as negative controls. Fluorescent images were captured using a Zeiss LSM710 confocal microscope and analyzed with ImageJ. In further experiments, the optimal IKVAV and/or peptide concentrations were used.

### 2.5 Endothelial Monolayer Formation on Hydrogel Surface Assay

The BBB barrier integrity is primarily induced by the formation of the endothelium. To mimic the in vivo setting, HAECs were seeded on top of the fabricated 4% PEG hydrogels. To assess the sole and cumulative effects of laminin-mimetic peptide IKVAV and fibronectin-mimetic peptide RGD on the activity of the ECs, PEG hydrogels were functionalized at the following concentrations: IKVAV-300 μM, RGD-600 μM, and IKVAV:RGD-300 μM. After seeding, the adherence of the HAECs and the establishment of the endothelial monolayer were evaluated using an inverted light microscope.

### 2.6 TEER Measurement

The evaluation of HAECs monolayer tightness was achieved through TEER measurement using STX2 electrodes and EVOM2 at days 1–5 postseeding.^15^ The TEER measurement reflects the flux of ions across the monolayer. The electrodes were positioned correctly, not angled, and slightly touching the bottom of the well, with the shorter probe placed inside the inner compartment of the inset and the longer probe placed outside the inset. The TEER readings of the samples were recorded in a consistent order, starting with the blank (8 μm pore-size), fabricated PEG hydrogels (8 μm pore-size), the second blank (0.4 μm pore-size), HAECs, NHAs coculture (0.4 μm pore-size), and ending with the HAECs monoculture (0.4 μm pore-size). Between each measurement, the electrodes were placed for 30 s in the conical containing PBS to prevent any discrepancies in the readings. To obtain the true resistance of a specific sample, the TEER reading of the blank was subtracted from the TEER reading of the sample and then multiplied by the surface area of the 12-well insert.

### 2.7 Permeability Assay

The permeability of the HAECs monolayer was assessed using two commonly used inert permeability tracers, EBA (high-molecular-weight protein marker) and Na–F (low-molecular-weight tracer). The flux of EBA and Na–F across the HAECs monolayer was determined on day 5 postseeding. On the media were removed, and samples were washed with PBS. Permeability assay buffer containing Na–F and 2% BSA mixed with Evan’s blue dye was added to the inner compartment of the insets. The inserts were then transferred into new 12-wells containing permeability assay buffer. Samples were collected from the lower compartments at 30, 60, 90, and 120 min and directly replaced with fresh permeability assay buffer. The concentrations of EBA that crossed the fabricated in vitro BBB hydrogel model were determined by measuring the absorbance of the collected aliquots with a Multiskan EX Spectrophotometer using a 630 nm optical filter. The corrected concentration was obtained and plotted. The concentration of Na–F that crossed the fabricated in vitro BBB hydrogel model was analyzed by measuring the fluorescence intensity of Na–F via a Tristar2 S LB 942 filter-based multimode plate reader using a fluorescent excitation and emission filter pair [Ex(*k*) = 485 ± 10 nm; Em(*k*) = 530 ± 12.5 nm]. The final concentration was obtained and plotted.

### 2.8 Immunofluorescence

To examine the formation of TJs between HAECs seeded on PEG hydrogels, immunohistochemical staining of ZO-1 was conducted on day 5 postseeding. The protocol involved fixing the cells with 4% PFA for 25 min and permeabilizing them with 100% methanol for 1 h. The samples were then washed twice with PBS and the blocking solution consisting of 3% BSA and 3% NGS was added for 1 h at room temperature, followed by incubating them with primary antibody (rabbit anti-ZO-1 antibody). The samples were then labeled with a secondary antibody (Alexa Fluor 488 goat antirabbit IgG) followed by DAPI staining. Fluorescent images were captured by using a Zeiss LSM710 confocal microscope.

### 2.9 Statistical Analysis

All statistical tests were performed using the GraphPad Prism software. When comparing more than two independent groups, the one-way ANOVA Tukey (HSD) posthoc test was used for parametric data. Two-way ANOVA Tukey (HSD) posthoc test was used when independent groups were studied against two variables. A *p*-value less than or equal to 0.05 was considered significant according to the following criteria: ns for *p*-value >0.05; * or # for *p*-value ≤0.05; ** or ## for *p*-value ≤0.01; and *** or ### for *p*-value ≤0.001.

## 3 Results and Discussion

### 3.1 Viability and Live/Dead Assay

**Figure 1:**
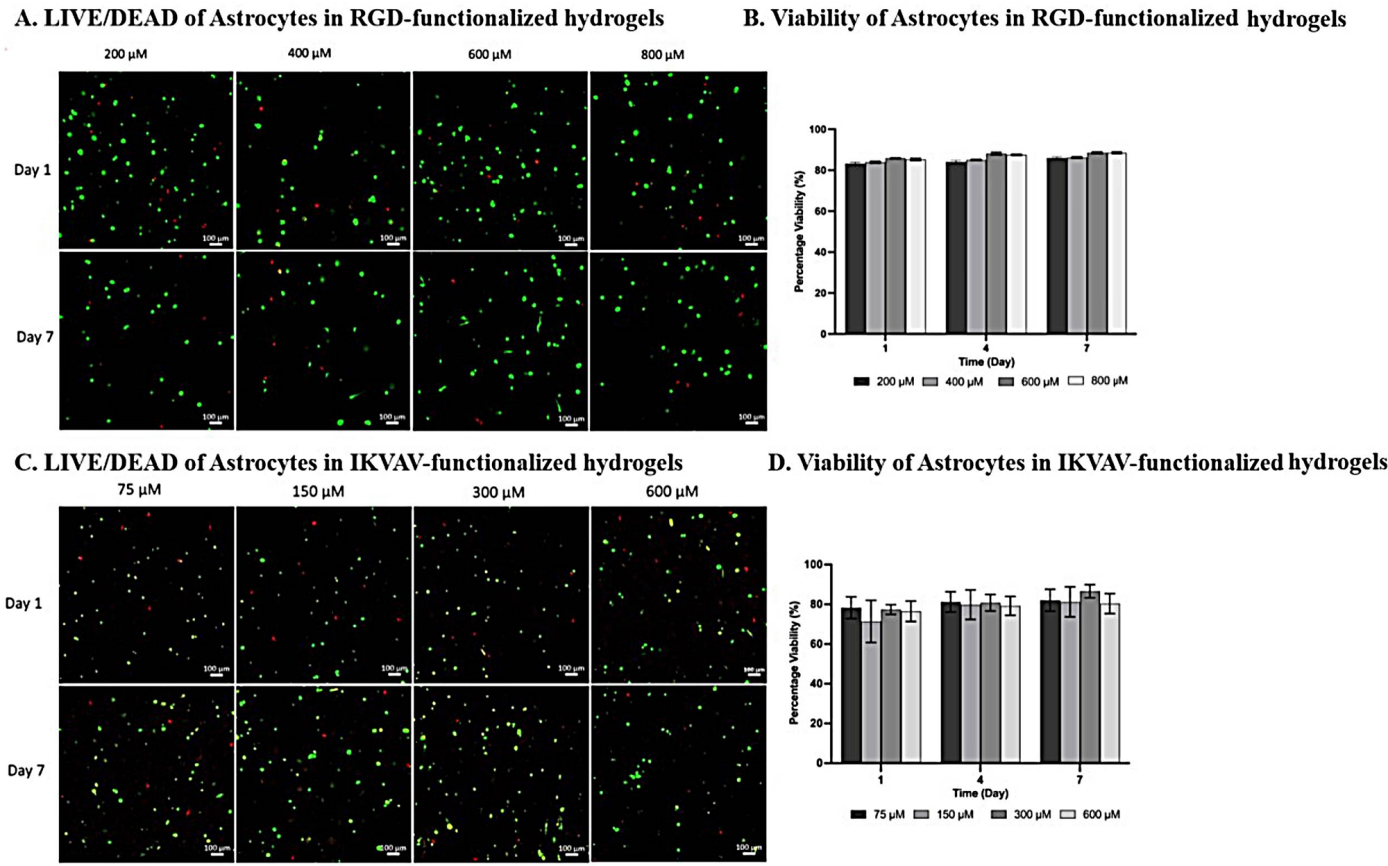
Live/dead assay and percentage viability of NHAs embedded in 4% PEG with different [A and B] RGD (200, 400, 600, and 800 μM) and [C and D] IKVAV concentrations (75, 150, 300, and 600 μM) at days 1, 4, and 7. Live NHAs were stained with Calcein-AM (green). Dead NHAs were stained with EthD-1 (red). Images were taken using a confocal microscope with a 10× objective. Scale bar = 100 μm. No statistical significance was found (*p*-value> 0.05). Error bars represent ± SEM (*n* = 3).

The ideal scaffold materials should be capable of not only connecting and supporting seeded cells but also maintaining the cell phenotype. In the initial assessment of hydrogel biocompatibility and cell support, the viability of NHAs cultured in functionalized 4% PEG hydrogels was examined; 4% PEG was selected based on a preliminary study (Supporting Information). The hydrogels were functionalized with varying concentrations of fibronectin-mimetic peptide RGD (200, 400, 600, and 800 μM) and laminin-mimetic peptide IKVAV (75, 150, 300, and 600 μM) over 7 days. Astrocytes remained highly viable in all PEG hydrogels, regardless of the concentrations of IKVAV and RGD, with viability consistently exceeding 80% at day 7 as depicted in Figure 1. Notably, there was no significant difference observed in viability across different concentrations or over time. This sustained viability is particularly significant given that unfunctionalized PEG, being an inert polymer, typically induces cell death over time.^16^ The extended survival of astrocytes in our study can be attributed to the incorporation of IKVAV and RGD into the hydrogels, underscoring the critical role of these peptides in supporting astrocyte viability within the 3D gel environment.^17^ Additionally, the use of PEG as a scaffold offers advantages such as biocompatibility, tunable properties, and inertness, making it an excellent platform for constructing complex 3D models of the BBB.^11^ Specifically, PEG’s hydrophilic nature provides an excellent environment for cell encapsulation and proliferation, while its tunable properties allow for precise control over mechanical stiffness, degradation rates, and biochemical functionality.^18^ Additionally, PEG’s inertness minimizes nonspecific interactions, reducing the likelihood of immune responses and facilitating long-term cell survival.^19^ Interestingly, despite peptide functionalization, there was a negligible effect on astrocyte proliferation. This limited proliferation aligns with previous research findings attributing such behavior to the constraints imposed by the 3D gel structure on cell growth.^8,20^ These results indicate that the RGD- and IKVAV-functionalized hydrogels are suitable for maintaining the viability of astrocytes.

The consistent viability of astrocytes, irrespective of the peptide concentration, highlighted the crucial role of astrocyte spreading in determining the optimal peptide concentration. Despite the overall high viability observed over 7 days, a subset of astrocytes exhibited a rounded morphology, especially when treated with IKVAV (Figure 1). This result aligns with previous findings indicating that RGD-functionalized hydrogels effectively promoted the spreading and branching of astrocytes compared to IKVAV.^20^ This phenomenon could be linked to the highly hydrophilic nature of PEG, which might limit the activity of the less hydrophilic IKVAV while attracting the more hydrophilic RGD, thereby masking IKVAV’s cell-promoting ability.^21^ Additionally, previous studies have noted rounded cell morphology in PEG hydrogels, possibly due to high cross-linking, which could obstruct integrin receptors and cell attachment sites.^16,22^ In line with these observations, our images demonstrated that 600 μM RGD emerged as the optimal concentration for promoting NHAs spreading. Conversely, the ideal IKVAV concentration was determined to be 300 μM based on the 3D morphology of astrocytes, consistent with prior research indicating 300 μM IKVAV as optimal for cellular differentiation in a 3D context.^23^

### 3.2 Endothelial Monolayer Formation on Hydrogel Surface Assay

**Figure 2:**
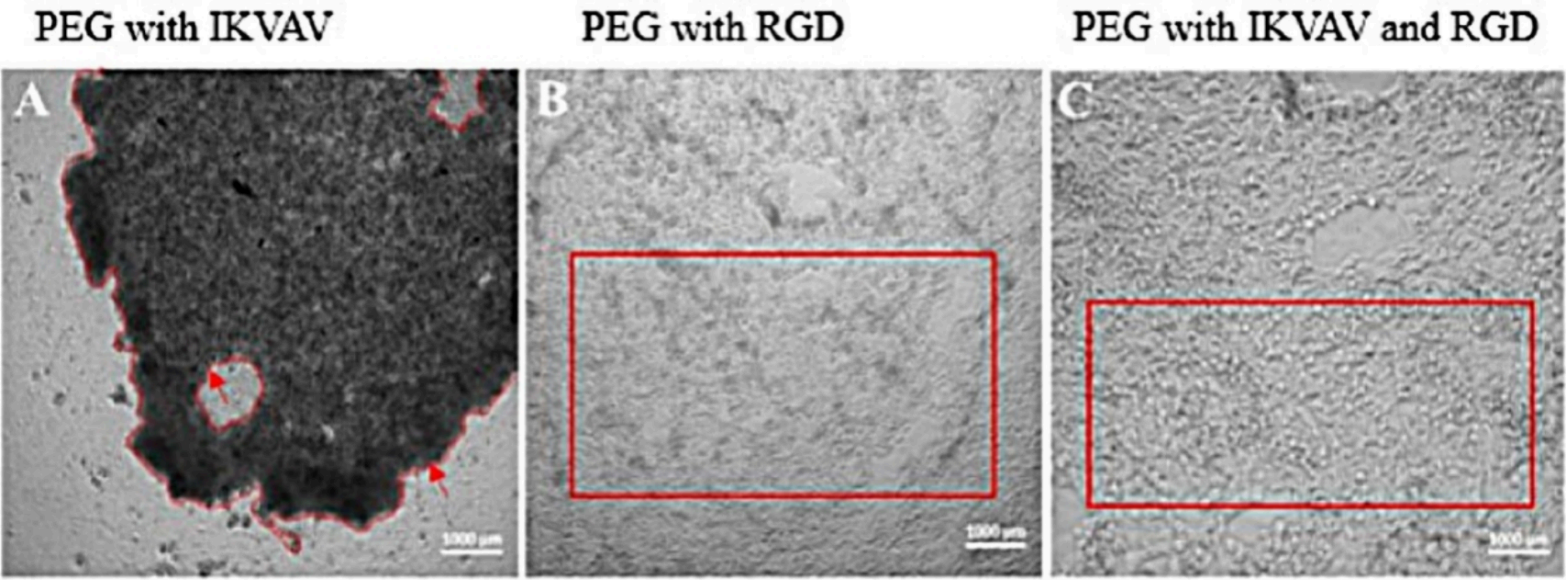
Microscopical images showing the morphological structure and the formation of a monolayer of the seeded HAECs on different functionalized PEG hydrogels. HAECs seeded on [A] 300 μM IKVAV-PEG-based hydrogel were unable to adhere and formed a clump (boarded in red), whereas HAECs seeded on [B] 600 μM RGD and [C] 300 μM IKVAV:RGD (1:1)-PEG-based hydrogels were able to form a monolayer and preserve their structures. Images were taken using an inverted light microscope. Scale bar = 1000 μm (*n* = 3).

In the subsequent phase, HAECs were seeded onto the surface of PEG hydrogels functionalized with either 600 μM RGD or 300 μM IKVAV. Following seeding, the adhesion of HAECs and the formation of an endothelial monolayer were meticulously assessed using an inverted light microscope. Notably, a well-defined monolayer was successfully established on the RGD-PEG hydrogels (Figure 2B). However, HAECs exhibited limited adhesion to PEG hydrogels functionalized with the IKVAV peptide (Figure 2A). This observed disparity can be attributed to the superior adhesion properties of fibronectin, particularly in the hydrophilic PEG hydrogel, in contrast to laminin.^24^ To overcome this challenge, a novel strategic approach was performed by combining IKVAV with RGD in a 1:1 ratio to enhance its signal within a PEG network and form a monolayer (Figure 2C).

### 3.3 TEER Measurement

**Figure 3:**
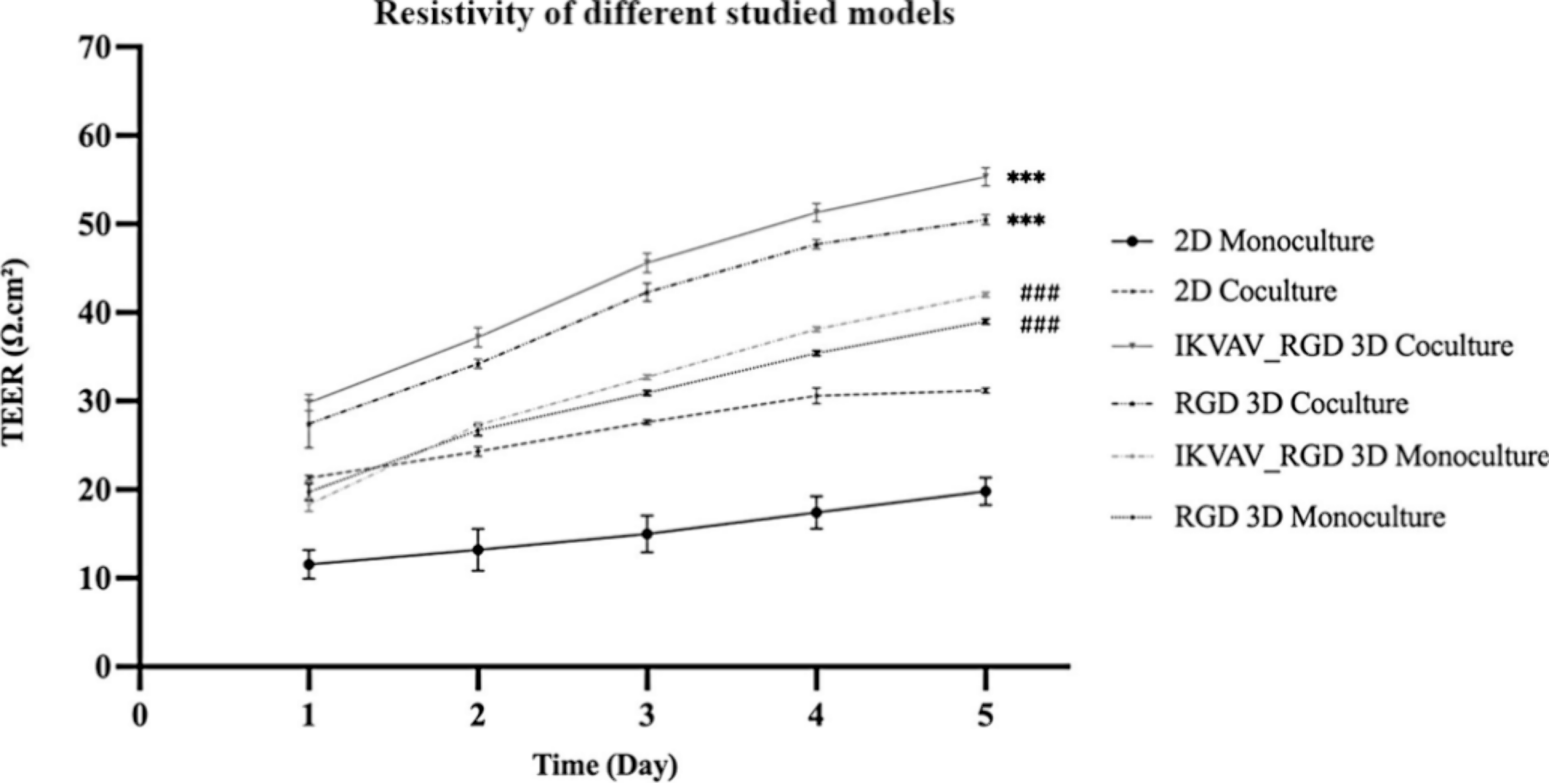
TEER values for different samples were recorded on days 1–5 using EVOM2 and STX2 electrodes. One-way ANOVA analysis was performed to determine the statistical significance between TEER at days 1 and 5 of each condition and between different samples at day 5. *p*-Value ≤0.05 was considered significant and marked with (* for hydrogel coculture model analysis and # for hydrogel monoculture model analysis). Error bars represent ± SEM (*n* = 3).

To evaluate the integrity of the barrier established by the monolayer, TEER was measured until a steady state was reached after 5 days, indicating the maximum formation of TJs.^25^ Notably, a significant increase in TEER was consistently observed across all conditions between days 1 and 5 (*p*-value <0.001) (Figure 3). In line with prior research, when HAECs were cocultured with NHAs in the presence of IKVAV and RGD-functionalized PEG hydrogel, the recorded TEER value was significantly higher than all other conditions (55.33 ± 1.47 Ω·cm^2^ at day 5; *p*-value <0.001; Figure 3).^26,27^ This was followed by the RGD cocultured hydrogel (50.46 ± 0.42 Ω·cm^2^ at day 5), and the monoculture hydrogel controls (IKVAV:RGD hydrogel: 42 ± 0.82 Ω·cm^2^ at day 5; RGD hydrogel: 39 ± 0.42 Ω·cm^2^ at day 5; *p*-value <0.001; Figure 3). This finding underscores the critical role of nonplanar dimensionality, cell–cell interactions, and cell–ECM interactions in enhancing the tightness of the barrier. These factors help maintain the cellular phenotype and facilitate the release of TJ proteins.^26^ Specifically, nonplanar dimensionality is crucial for enabling astrocytes to maintain their phenotype and release vital secretions that promote TJ protein release.^28^ Additionally, PEG’s inertness reduces the likelihood of disruption to cell–cell junctions, thereby contributing to the resistance.^19,29^ The hydrophilic nature of PEG further contributed to the establishment of a suitable microenvironment for cell adhesion and barrier formation, therefore promoting TJ formation between endothelial cells.^18^ However, the maximum TEER obtained from our model is considerably lower than the in vivo TEER, which is estimated to exceed 1000 Ω·cm^2^.^30^ This discrepancy can be attributed to the use of immortalized endothelial cells, which typically form only limited restrictive monolayers in vitro, resulting in TEER values ranging from 20 to 200 Ω·cm^2^.^31^ Despite this limitation, in vitro models utilizing human-immortalized cell lines offer several advantages, especially during the initial phases of research.

### 3.4 Permeability Assay

**Figure 4:**
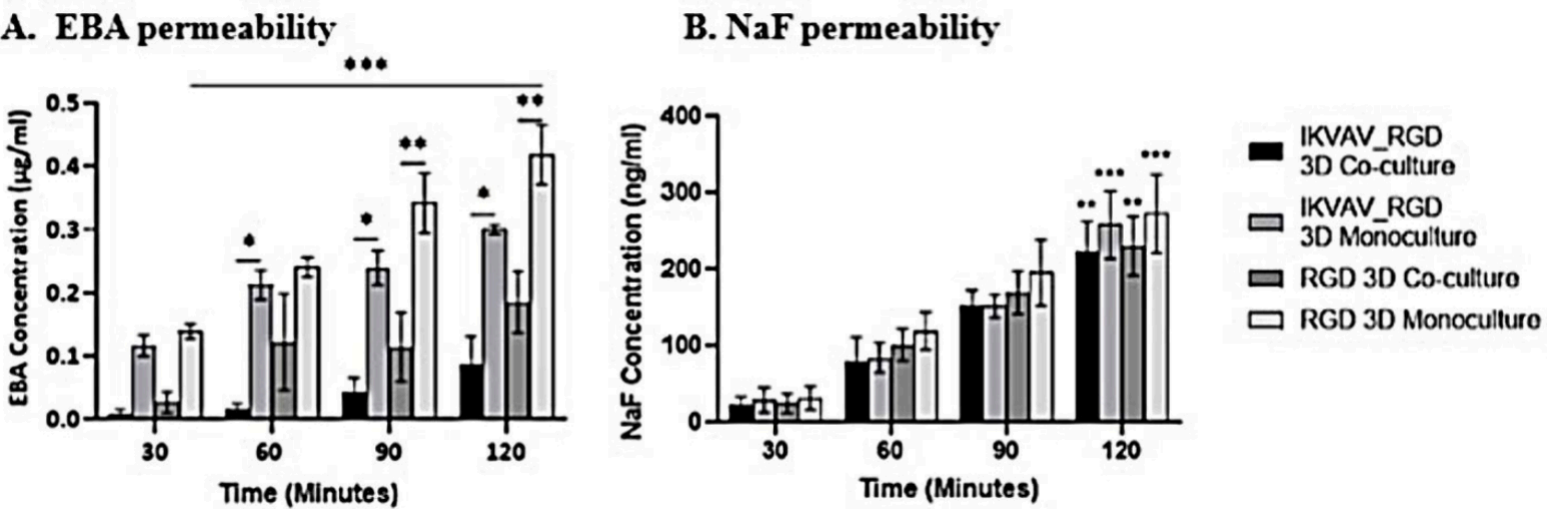
[A] EBA and [B] Na–F permeability assay: Log10 concentration of the Evan’s blue bound to albumin (EBA; μg/mL) and sodium fluorescein (Na–F; ng/mL) under different functionalized PEG conditions at four time-points (30, 60, 90, and 120 min). Two-way ANOVA analysis was performed to determine any statistical significance. *p*-Value ≤0.05 was considered significant and marked with (*) for overtime analysis or (#) same-time point analysis. Error bars represent ± SEM (*n* = 3).

Following the TEER assessments, permeability assays were conducted on day 5 using low-molecular-weight Na–F and high-molecular-weight EBA tracers. Results in Figure 4 demonstrated a significant increase in EBA permeability for all conditions at this extended time frame (*p*-value <0.001). Notably, the IKVAV:RGD coculture hydrogel exhibited the lowest permeability (0.216 ± 0.055 μg/mL at 120 min). Remarkably, the EBA concentration was significantly lower in the coculture model compared to their respective monoculture controls (*p*-value = 0.0144 for RGD hydrogel and *p*-value = 0.0329 for IKVAV:RGD hydrogel; Figure 4), and in 3D models compared to their counterparts (*p*-value <0.05). This underscores the pivotal role of astrocytic endfeet interactions in a 3D environment with other molecules, leading to a reduction in BBB permeability.^32^ Our findings align with existing research indicating the crucial roles of laminin and fibronectin in maintaining BBB permeability, where disruption of these vital cell–cell or cell-matrix interactions has been shown to increase permeability.^33^ The Na–F permeability assay, assessing the paracellular flow of small polar molecules across the endothelium, supported our observations. As expected, Na–F demonstrated relatively higher permeability over time compared to EBA but was still more restricted in our model, consistent with the BBB’s ability to allow the relative paracellular flow of small molecules (Figure 4).^34^ Importantly, our results highlight that models exhibiting the highest TEER also show the least permeability.

### 3.5 Immunofluorescence Assay

**Figure 5:**
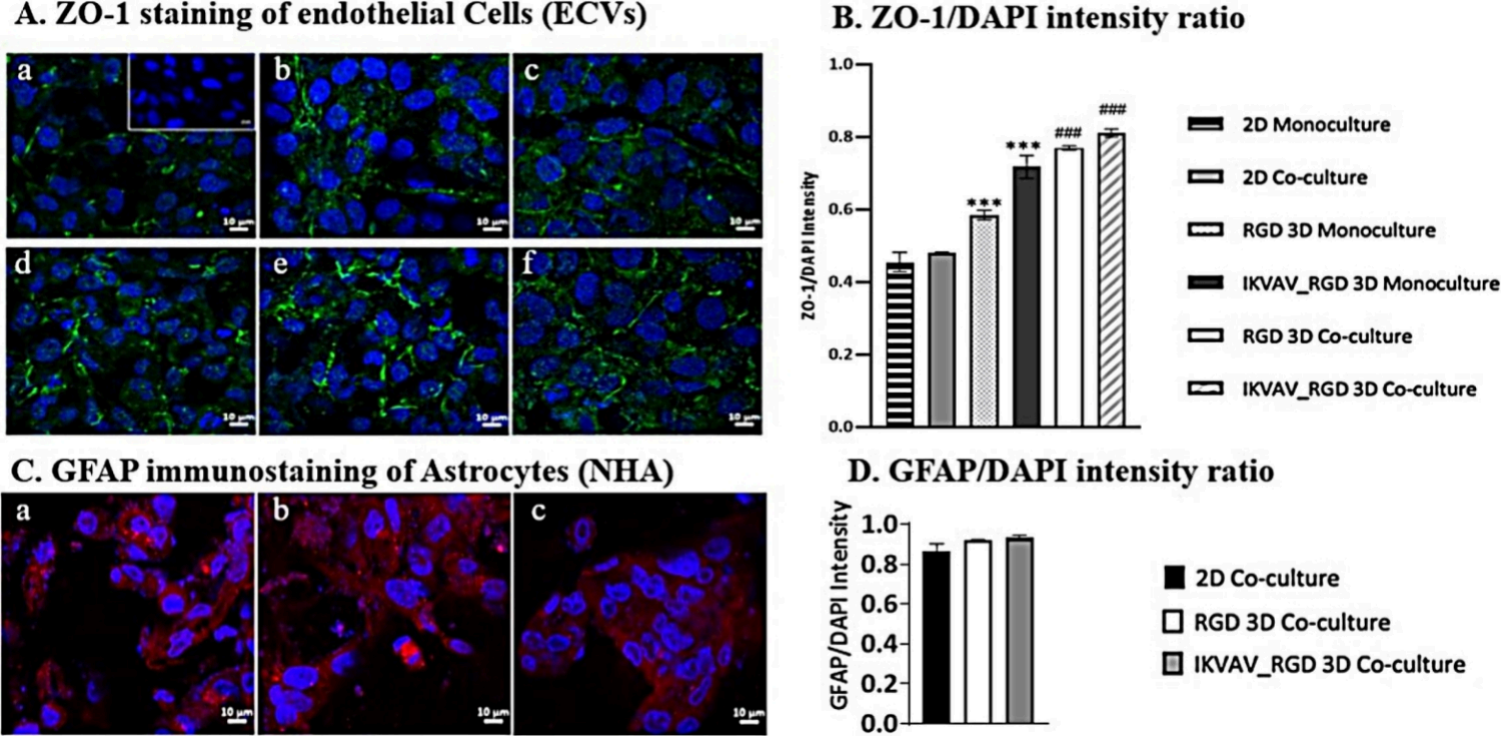
[A] ZO-1 staining of HAECs in (a) HAECs monoculture control; negative control, (b) HAECs and NHAs coculture control, (c) RGD-600 μM control, (d) IKVAV:RGD-300 μM control, (e) RGD-600 μM with encapsulated astrocytes, (f) IKVAV:RGD-300 μM with encapsulated NHA. [B] The ZO-1/DAPI intensity ratio of each condition was quantified using ImageJ. [C] GFAP immunostaining of NHA in (a) IKVAV:RGD-300 μM with encapsulated NHA, (b) RGD-600 μM with encapsulated astrocytes, (c) HAECs and NHAs cocultured on insert. [D] GFAP/DAPI intensity ratio of each condition quantified using ImageJ. Images were taken using a confocal laser scanning microscope (63×). Scale bar = 10 μm. *p*-Value ≤0.05 was considered significant and marked with (* for monoculture samples and # for coculture samples). Error bars represent ± SEM (*n* = 3).

The barrier properties of this model were further investigated using immunofluorescence staining for ZO-1 TJ protein and quantified by calculating the ZO-1/DAPI ratio. All hydrogels, particularly the IKVAV:RGD coculture hydrogel, exhibited significantly higher ZO-1 expression compared to 2D controls (*p*-value <0.001; Figure 5A,B). However, none of the conditions managed to establish a continuous ZO-1 protein network along the borders. This observed discontinuity can be attributed to the use of immortalized endothelial cells, which not only fail to replicate the in vivo BBB endothelial cell phenotype but may also exhibit pathological properties of the BBB.^35^ Additionally, the absence of a continuous network can be linked to the lack of other essential basement membrane molecules and cells, such as collagen-IV and pericytes These missing elements are crucial in the intricate interplay within our cellular and molecular environment, enhancing the expression of TJ proteins, including ZO-1.^36^ For example, TGF-β released by the astrocytic endfeet, after interacting with pericytes in vivo, has been shown to increase the expression of TJ proteins, such as claudin-5.^37^ Regardless, the low GFAP expression in astrocytes reflects the NHA’s are not in a pathological state and are capable of interacting via their endfeet with neighboring HAECs, IKVAV, and RGD to enhance the expression of ZO-1 (Figure 5C,D).^38^

## 4 Conclusions and Outlook

This study presents a novel in vitro model of the BBB that utilizes a PEG hydrogel cocultured with astrocytes and endothelial cells, and functionalized with laminin and fibronectin-mimetic peptides, IKVAV and RGD, respectively. Our evaluation of this model’s barrier properties reveals promising outcomes, suggesting its potential as a biomimetic BBB in vitro system for neurological disease studies and reliable drug delivery assessments. Our findings underscore the significance of establishing a 3D microenvironment that incorporates crucial cell–cell and cell–ECM interactions to enhance barrier integrity. Specifically, our model demonstrates the critical role of astrocytes grown within a 3D environment, combined with the presence of both IKVAV and RGD, in achieving an enhanced cellular morphology, higher TEER, increased ZO-1 expression, and a more restricted and selective permeability. Further work is still needed to enhance the features of the current model. This includes enabling the incorporation of shear stress for a more realistic representation of the in vivo microcirculatory environment in the brain, as well as integrating collagen-IV mimetic peptide GFOGER and pericytes into our model to assess their individual and collective impact on BBB integrity. These modifications will allow the model to offer significant insights into BBB physiology and pathology, aiding in the development of novel drugs and successful delivery strategies.

## Supporting Information Available

Additional experimental details, methods, and results, including figures for the mechanical and chemical testing of the hydrogel (S1 and S2) (PDF)
