# Supplementary material for: Engineering a 3D Biomimetic Peptides Functionalized-Polyethylene Glycol Hydrogel Model Cocultured with Endothelial Cells and Astrocytes: Enhancing In Vitro Blood–Brain Barrier Biomimicry
Source: Mol Pharm. 2024 Aug 12;21(9):4664–72. doi: 10.1021/acs.molpharmaceut.4c00599 (PMC11372828; doi:10.1021/acs.molpharmaceut.4c00599)
Supplement: Supplementary file 1 — mp4c00599_si_001.pdf [file mp4c00599_si_001.pdf]

Engineering a 3D Biomimetic Peptides Functionalized-Polyethylene Glycol Hydrogel Model Co-cultured with Endothelial Cells and Astrocytes: Enhancing In Vitro Blood-Brain Barrier Biomimicry

Nesrine Ahmad[a], Georges Kiriako[a], John Saliba[a, b], Kawthar Abla[a], Marwan El-Sabban[b*], Rami Mhanna[a*]

[a] *Biomedical Engineering Program, Maroun Semaan Faculty of Engineering and Architecture, American University of Beirut, Beirut, 1107 – 2020, Lebanon*

[b] *Department of Anatomy, Cell Biology and Physiological Sciences, Faculty of Medicine, American University of Beirut, Beirut, 1107 – 2020, Lebanon*

*****E-mail: rm136@aub.edu.lb

*E-mail: me00@aub.edu.lb

Methods

1. Mechanical characterization of the hydrogels prepared by different PEG concentrations

The Different concentration of PEG hydrogel was characterized regarding their mechanical strength (hardness) using Young's moduli. Compressive moduli of the swelled hydrogels were measured using a texture analyzer (Instron 5900 series, US). Samples were compressed at a speed of 0.01mm/s and Young's moduli will be calculated according to Hooks Law:

$F=-kx$

Where F is the force, x is the length of extension/compression, and k is a constant of proportionality known as the *spring constant* which is usually given in N/m.

2. Live/Dead cells assay of the hydrogel prepared by different PEG concentrations

To select the optimal PEG concentration, cell viability of astrocyte cells cultured in the PEG hydrogel was assessed by using Live/Dead assay. The cells were stained with Calcein AM and ethidium Homodimer-1 (EthD1). Hydrogels were washed 3 times with PBS 1X and subsequently incubated in a solution containing 2 µM Calcein AM and 4 µM EthD-1 for 30 minutes at 37 °C. Constructs will then be washed three times with PBS 1X and imaged directly with a Zeiss LSM710 confocal microscope at five different focal points.

Results

The stiffness of different PEG hydrogel concentrations without peptide modifications was studied. The stiffness ranged from 4 to 15 KPa which is similar to that of the brain **(Fig. S1).**

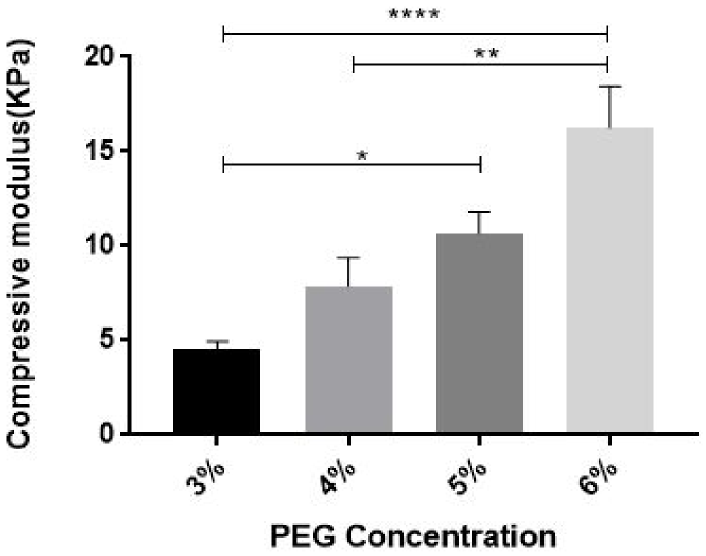

Fig S1. Compressive modulus of PEG at different concentrations.

The growth of normal human astrocytes (NHAs) embedded in different PEG concentrations without peptide modification was then evaluated. PEG hydrogels showed 10% cell viability at all concentrations on all days. When modified with 100 µM RGD (fibronectin peptide), cell viability increased significantly ($p<0.001$) to 90% for all concentrations at day 1 but only 3% and 4% PEG maintained this viability at day 7. Astrocytes were circular, small, and non-proliferative which indicates that they were in their native physiological state (**Fig S2**).

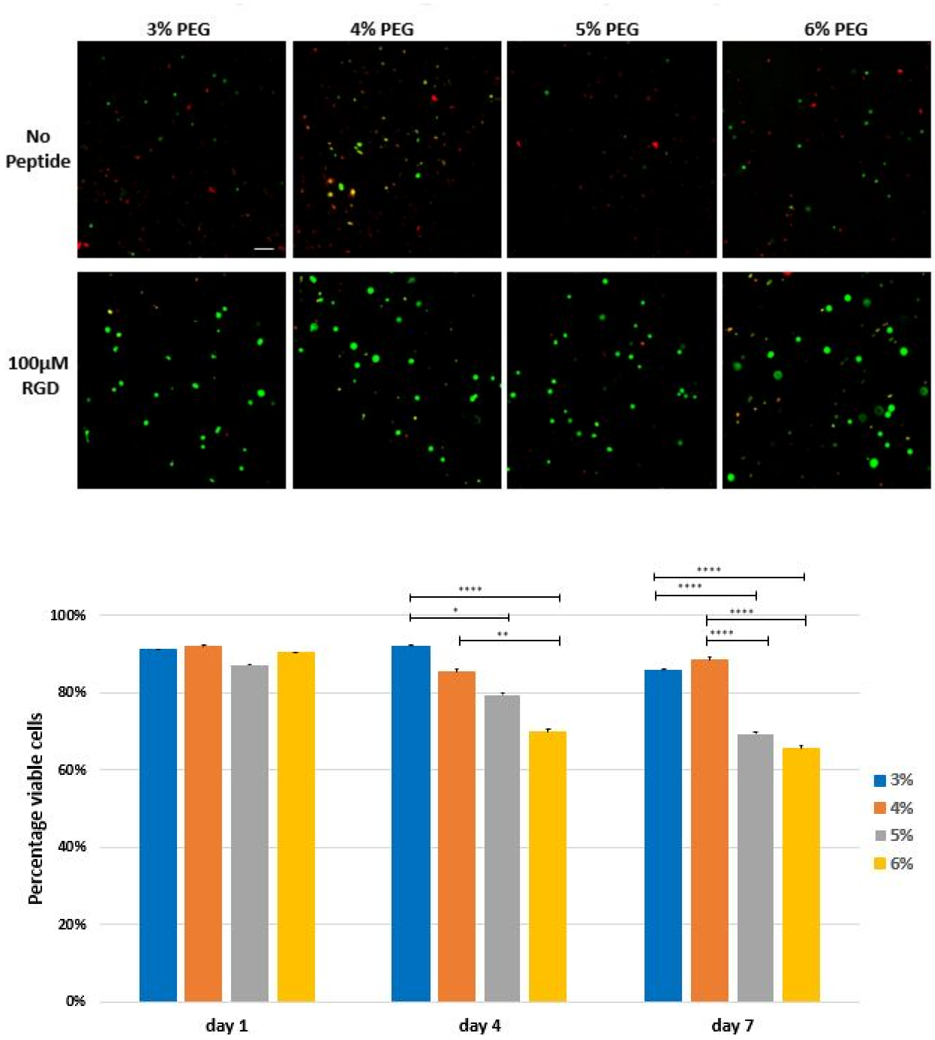

Fig S2. Live/dead assay and percentage viability of NHAs embedded in different concentrations of PEG hydrogels on days 1, 4, and 7. Images above show live (green)/dead (red) cells encapsulated for 7 days in different gel conditions. Images were acquired using a confocal microscope with a 10X objective. Statistical differences were determined using two-way ANOVA and Tukey tests. P values of less than 0.05 were considered statistically significant (*), with $P \leq 0.01$ represented with **, and $P \leq 0.001$ represented with ***.

Based on the above results, 4% PEG hydrogel was selected for further studies.
